# Dynamic assessment of variations in pupil diameter using swept-source anterior segment optical coherence tomography after phakic collamer lens implantation

**DOI:** 10.1186/s40662-021-00262-2

**Published:** 2021-10-24

**Authors:** Felix Gonzalez-Lopez, Carmen Bouza-Miguens, Victor Tejerina, Vasyl Druchkiv, Blas Mompean, Julio Ortega-Usobiaga, Rafael Bilbao-Calabuig

**Affiliations:** 1 Department of Refractive Surgery, Clinica Baviera, Melchor Fernández Almagro, 9, 28029 Madrid, Spain; 2 Department of Research and Development, Clinica Baviera, Valencia, Spain

**Keywords:** Phakic collamer lens, ICL, Pupil, OCT, Central hole, Central port, Posterior chamber phakic lens

## Abstract

**Purpose:**

To dynamically assess variations in pupil diameter induced by changes in brightness in myopic eyes implanted with an implantable collamer lens (ICL, STAAR Surgical) with a central port.

**Methods:**

This prospective, observational single-center case series study comprised 65 eyes from 65 consecutive patients undergoing ICL implant. A modified commercially available swept-source Fourier-domain anterior segment optical coherence tomography (AS-OCT) device was used for imaging and performing dynamic pupillometry under changing light conditions before and after a mean follow-up interval of four months after surgery.

**Results:**

Preoperative mean pupil size under photopic conditions was 3.38 ± 0.64 mm; after surgery, this increased to 3.48 ± 0.61 mm. Mean pupil size under scotopic light conditions was 5.72 ± 0.79 mm before surgery and 5.84 ± 0.77 mm postoperatively. The differences between preoperative and postoperative pupil diameter in miosis and mydriasis were 0.10 ± 0.44 mm (*P* = 0.078) and 0.12 ± 0.58 mm (*P* = 0.098), respectively. The scotopic pupil exceeded the optic zone of the implanted lens in 39 eyes (60%). The mean central vault value was 412 ± 177 μm under maximum miosis and 506 ± 190 μm under maximum mydriasis. We found a positive correlation between vault and differences in pupil diameter under all light conditions (*P* < 0.05).

**Conclusion:**

Dynamic AS-OCT enables a very precise determination of the pupillary diameter in the iris plane. The changes in the pupil diameter under different light conditions after the implantation of an ICL are related to the postoperative vault.

**Supplementary Information:**

The online version contains supplementary material available at 10.1186/s40662-021-00262-2.

## Background

In the last two decades, the implantable collamer lens (ICL) (STAAR Surgical Company, Monrovia, CA, USA) has become widely accepted as a useful option for the surgical correction of various refractive defects [1–3].

Advances in myopic lens design in 2011 with the development of a central port measuring 0.36 mm in diameter (ICMV4c model) have simplified surgery and reduced postoperative complications, such as pupillary block and the formation of anterior subcapsular cataracts [1, 4–7]. However, in some cases, dysphotopsia, understood as an unwanted image that patients perceive after posterior chamber phakic intraocular lens (pIOL) surgery, may remain.

One kind of linear dysphotopsia, reported by some patients in their lower visual field, was associated with peripheral laser iridotomies or intraoperative iridectomy [8], which were necessary during implantation of previous models without a central port. Since these maneuvers are no longer required with the advent of new models, this sort of dysphotopsia has now disappeared. However, drilling the center of the optical zone has repercussions, and new dysphotopsias associated with the central port have been reported [9, 10]. This type of dysphotopsia may occur alongside another type that is perceived by some patients, especially under scotopic light conditions, and that originates as a result of a mismatch between the pupil diameter in mydriasis and the size of the optical zone of the pIOL: if the diameter of the pupil exceeds the boundaries of the optical zone, then two different images could be generated on the retina [11, 12]. Patients complain of halos under these circumstances, and sometimes even in mesopic ambient light e.g., during the use of electronic devices, which produces a ghost image.

Attempting to resolve this problem, a new model of ICL (EVO +) with an enlarged optical zone was developed in 2016. The lens is available in powers of up to − 14.5 diopters (D). Even so, the pupils mainly of young myopic patients could exceed the optical zones in the dark [13].

Therefore, when considering the surgical indication for a pIOL, it is of paramount importance to estimate the possibility that patients may experience night vision disorders. In this sense, if we know the preoperative scotopic pupil size at the iris plane and the optical zone diameter of the pIOL to be implanted as a function of refraction, we could easily identify patients at risk of experiencing visual disturbances due to the pupil exceeding the optical zone of the pIOL. However, the ICL is implanted in the posterior chamber, where it remains in continuous and direct contact with the back surface of the iris, and thus potentially leading to a disturbance in pupil diameter and movements. Studies addressing these changes in pupillary features after implantation of an ICL report contradictory outcomes [12, 14–19].

The aim of this study was to describe changes in pupil diameter after implantation of a central-hole ICL. To do so, we designed a precise pupillometry technique based on dynamic anterior segment optical coherence tomography (AS-OCT).

## Patients and methods

We performed a prospective observational single-center study of eyes undergoing implantation of an ICL to correct myopia and myopic astigmatism. The sample for this trial comprised 65 eyes (29 right) from 65 consecutive patients (42 females) who underwent uneventful implantation of spherical (52 eyes) and toric (13 eyes) central hole pIOLs (Visian ICL EVO/EVO + models) for correction of myopia. Only the first operated eye was enrolled. All operations were performed at Clinica Baviera (Madrid, Spain) by the same experienced surgeon (FGL). All surgeries were uneventful, and no complications occurred during the follow-up period.

All patients gave their written informed consent for the surgical procedure and for the use of their personal data in medical and scientific research. Data collection fulfilled Spanish legal requirements, and the study was performed in accordance with the requirements of the Medico-Legal Committee of Clinica Baviera.

### Assessment of pupil diameter and vault using AS-OCT

The latest commercially available 3-dimensional swept-source Fourier-domain AS-OCT device (CASIA2, Tomey Corp, Nagoya, Japan) was used for imaging and dynamic pupillometry under changing light conditions. We used a specific beta version software application for the dynamic assessment of pIOLs; the term “dynamic” referred to the changes that occur in the structures of the anterior segment induced by modifications in external ambient luminance. The new built-in software video system runs at a very high scanning speed (20 frames per second) and provides high-resolution images. It also automatically controls the illumination from a light-emitting diode source which appears in the anterior segment movie mode in order to create different levels of lighting exposure. We regulated the intensity of the light to the current maximum of 990 lx, as measured using a PCE-MLM1 Light Meter (PCE Instruments, Meschede, Germany). The study eye was focused on an internal dot during imaging, and a 15-s video recording was started under dark ambient illumination (0.5 lx) in the examination room, waiting one minute for the patient to adapt to the dark conditions before starting the test. After 5 s, the light was turned off for 5 s, and the recording continued for a further 5 s, once again under scotopic ambient light. An additional movie file shows the methodology used in more detail (see Additional file 1).

**Fig. 1:**
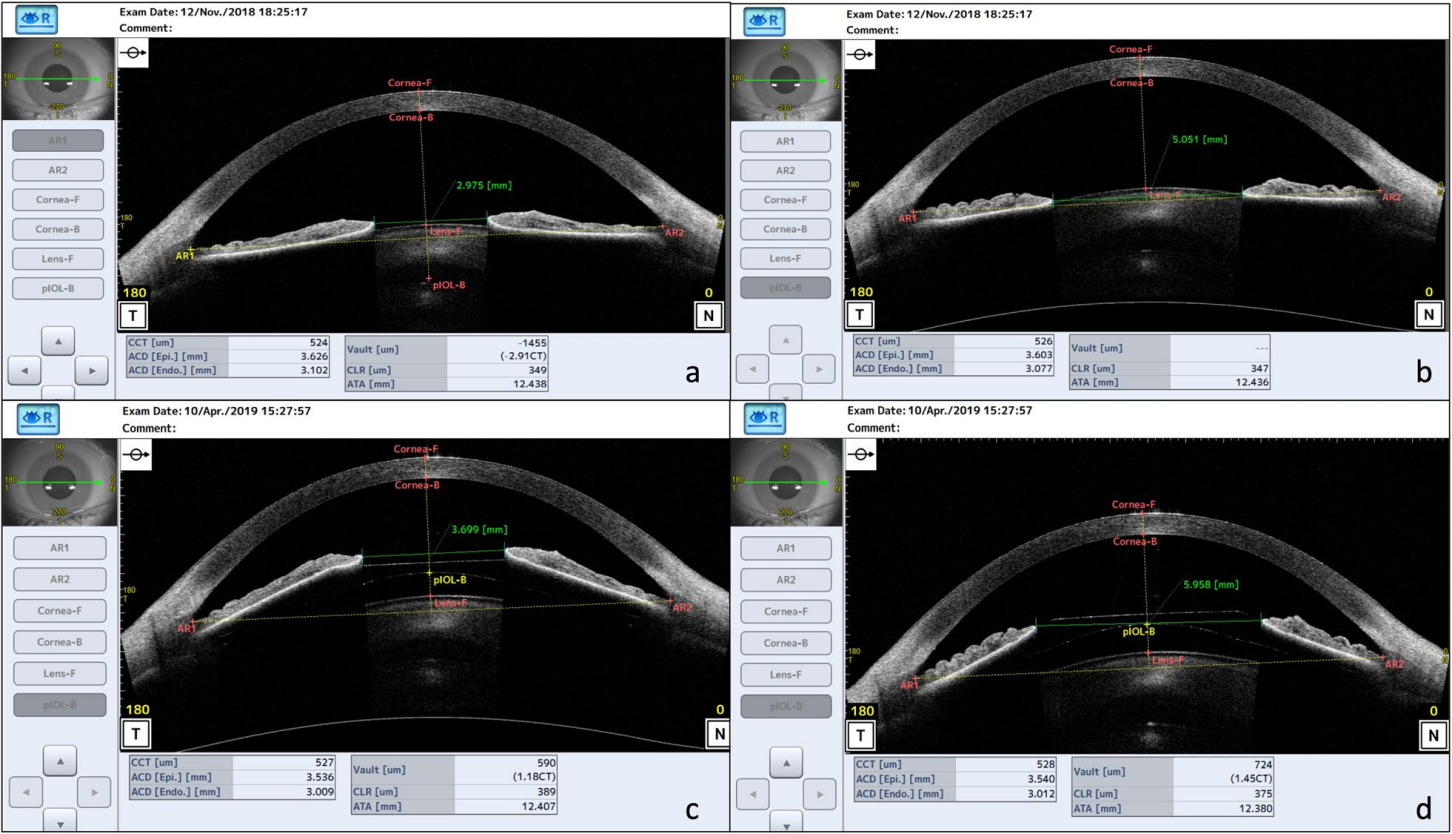
Anterior segment optical coherence tomography showing pupil and vault measurements in miosis and mydriasis, before (**a**, **b**) and after implantable collamer lens implantation (**c**, **d**)

Images at the minimum and maximum pupil size were manually selected. Iris distance, measured in the horizontal meridian in the iris plane from the nasal and temporal inner border of the iris, was recorded in all selected OCT frames before surgery and around 4 months postoperatively. Vault was also gauged after surgery (Fig. 1). We calculated the vault range, defined as the absolute difference between the two central vault interval values measured in mydriasis and miosis [20].

### Validation of the internal light of the AS-OCT

Prior to our study, we validated the internal light of the CASIA software. To do so, we analyzed the inter-rater and intra-rater reliability of AS-OCT pupil diameter measurements. Two blinded examiners analyzed the same 20 measurements from 10 patients. Three OCT video measurements were recorded for each subject in intervals of 15 min under the same ambient light conditions, as described above, to obtain OCT frames in maximum mydriasis and miosis for each eye.

The overall mean rating of both examiners in miosis was 3.26 mm. The inter-eye standard deviation (SD) was 0.32 mm, and the intra-eye SD was 0.18 mm (repeatability coefficient = 0.49 mm; coefficient of variation = 5.4%). The SD attributed to the 2 raters was 0.05 mm (reproducibility coefficient = 0.131 mm; coefficient of variation = 1.5%).

In mydriasis, the overall mean rating was 5.51 mm. The inter-eye SD was 0.42 mm, and the intra-eye SD was 0.16 mm (repeatability coefficient = 0.45 mm; coefficient of variation = 3%). The SD attributed to the raters was 0.08 mm (reproducibility coefficient = 0.23 mm; coefficient of variation = 1.5%).

### Statistical considerations

The outcomes obtained from the OCT imaging analysis were entered into an Excel spreadsheet (Microsoft Corp, Redmond, Washington, USA). Data were analyzed using R Core Team (2019, R Foundation for Statistical Computing, Vienna, Austria). The results are expressed as mean ± SD for normally distributed data and medians and interquartile ranges (IQR) for non-normally distributed data. The differences were considered statistically significant when the *P* value was less than or equal to 0.05.

For the analysis of repeatability and reproducibility, we applied ANOVA under random factorial design, as discussed in Gwet et al.’s study [21]. We then calculated the repeatability coefficient i.e., the limit up to which 95% of all differences are likely to be included. We also calculated the reproducibility coefficient and reported the coefficient of variation, which was calculated as the quotient between the intra-eye SD and the overall mean for repeatability and between the rater SD and overall mean for reproducibility.

We compared differences in pupil diameter before and after surgery using a paired *t*-test. We used regression models, ordinary least squares method and Huber estimation method, to investigate the correlation between pupil diameter and vault value, age, lens power, ICL size, preoperative spherical equivalent and crystalline lens rise.

## Results

The preoperative mean age of the patients was 32 ± 7 years (range 21 to 48 years). The mean baseline preoperative spherical equivalent was − 9.46 ± 3.38 D (range − 3.00 to − 19.88 D). Lens size was distributed as follows: 12.1 mm in 2 eyes, 12.6 mm in 20 eyes, 13.2 mm in 40 eyes, and 13.7 mm in 3 eyes.

Mean pupil size under photopic conditions was 3.38 ± 0.64 mm (range 2.18 to 5.03 mm) before surgery, and 3.48 ± 0.61 mm (range 2.14 to 5.14 mm) after surgery. Mean pupil size under scotopic light conditions was 5.72 ± 0.79 mm (range 3.68 to 6.88 mm) before surgery and 5.84 ± 0.77 mm (range 3.88 to 7.04 mm) after surgery.

**Fig. 2:**
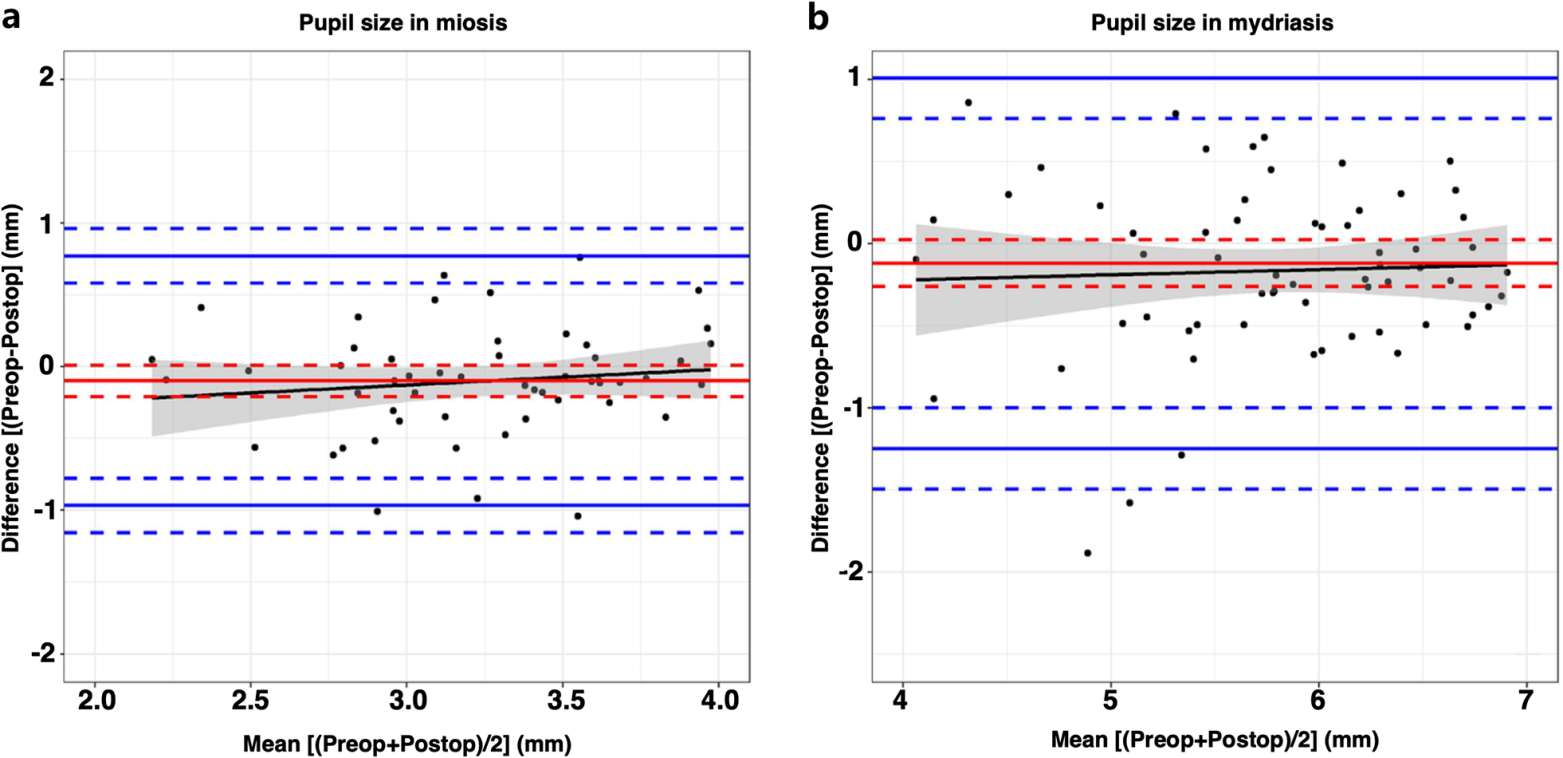
Differences in pupil diameter in miosis (**a**) and mydriasis (**b**) between the preoperative and the postoperative of the phakic surgeries

Differences in diameter between the preoperative and the postoperative pupil at a mean follow-up interval of 4 months after surgery [133 ± 55 days, (range 40 to 305 days)] in miosis and mydriasis were 0.10 ± 0.44 mm (range − 1.55 to 1.04 mm) and 0.12 ± 0.58 mm (range − 1.58 to 1.89 mm), respectively. Although we observed larger diameters under both ambient light conditions after surgery, we did not find statistically significant differences in miosis and mydriasis (*P* = 0.078 and *P* = 0.098, respectively; Fig. 2).

The same or a smaller postoperative pupil diameter was detected in miosis for 24 eyes [36.92%, median (IQR) difference preop-postop: − 0.17 mm (range − 0.48 to − 0.06 mm)] and in mydriasis for 25 eyes [38.46%, median difference preop-postop: − 0.30 mm (range − 0.58 to − 0.14 mm)]. The scotopic pupil exceeded the optic zone of the implanted lens by at least 0.1 mm in 39 eyes (60%). The mean scotopic pupil size of this group of eyes was 6.26 ± 0.50 mm (range 7.04 to 5.07 mm). In 18 eyes (46.15%), the pupil margin outreached the optic zone only on the nasal side, whereas for 21 eyes (53.85%) it exceeded both on the nasal and temporal sides. In no eyes the optical zone was exceeded only on the temporal side.

**Fig. 3:**
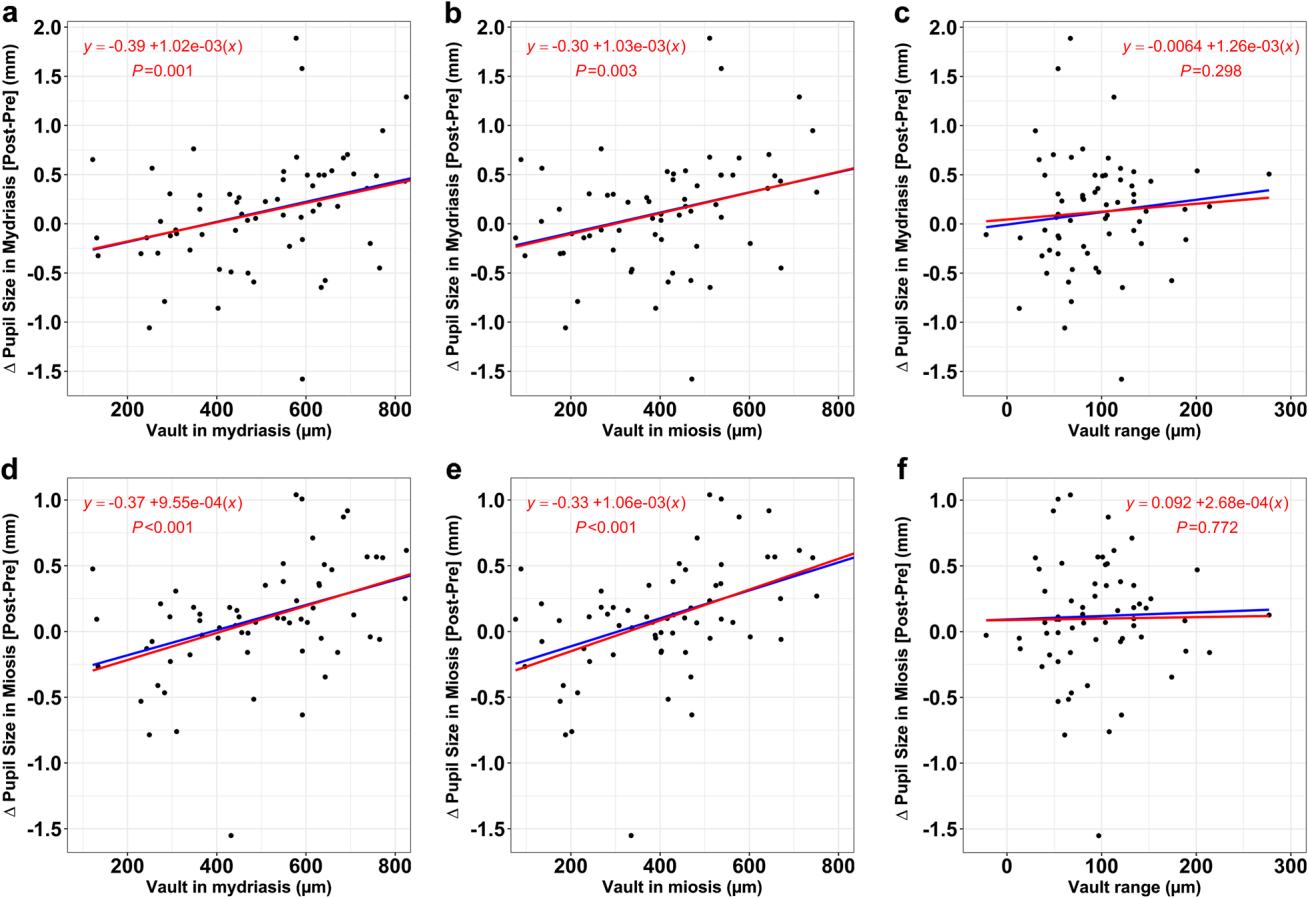
Correlation between change in pupil diameter in mydriasis (**a**, **b**) and in miosis (**d**, **e**) after implantable collamer lens implantation with the central vault postoperative measures, and with the vault range (**c**, **f**)

The mean central vault value was 412 ± 177 μm (range 76 to 845 μm) under photopic light conditions and 506 ± 190 μm (range 122 to 903 μm) in maximum mydriasis. The mean vault range was 95 ± 51 μm (range 13 to 277 μm). The scatter plots in Fig. 3a, b, d and e show the differences in pupil diameter under different light conditions with central vault values. We observed a positive correlation between vault and differences in pupil diameter under all light conditions (*P* < 0.05). Vault range does not seem to be related to differences in diameter (*P* = 0.298 in miosis, *P* = 0.772 in mydriasis; Fig. 3c and f).

We did not find any significant correlations between crystalline lens rises and pre- and post-operative changes in pupil diameter (*P* = 0.298 in miosis, *P* = 0.525 in mydriasis). Neither postoperative pupil size was significantly correlated to implanted lens power (*P* = 0.162 in miosis, *P* = 0.122 in mydriasis), ICL size (*P* = 0.28 in miosis, *P* = 0.31 in mydriasis) or preoperative spherical equivalent (*P* = 0.436 in miosis, *P* = 0.717 in mydriasis). Similarly, no significant correlation between age and pupil in miosis (*P* = 0.241) or mydriasis (*P* = 0.334) was found.

## Discussion

A standardized measurement of pupil size under defined light conditions with good repeatability is of the utmost importance in preoperative and postoperative evaluation of a patient candidate for phakic lens implant. Our study describes a new precise pupillometry technique based on dynamic AS-OCT and its clinical application in eyes that are candidates for implantation of a pIOL. Under different ambient light conditions (scotopic and photopic), we observed wider pupil sizes at a mean of four months of postoperative follow-up compared to preoperative values, although these differences were not statistically significant. Likewise, the correlation between postoperative vault and the increase in pupil size after the insertion of an ICL was significantly positive—the greater the vault, the greater the increase in pupil diameter—under all external light conditions. Despite this tendency of the pupil size to increase after the intervention, in some cases this did not happen, and the pupil diameter actually decreased. This observation reflects the contradictory results reported in the literature: though pupillary dynamics has been addressed in eyes implanted with an ICL, it has not yet been fully elucidated.

Keuch et al. [14] were the first to assess pupil constriction in eyes implanted with an ICL. The authors used a pupillograph to study 15 myopic eyes before and after implantation of an ICL without a central port (model V4). Approximately two weeks after implantation, pupil diameter was significantly smaller than before surgery (*P* < 0.01). Similar results were reported by Chun et al. [15], who found a significant decrease in pupil diameter 1 and 3 months after surgery; the diameter returned to the preoperative values at 6 and 12 months. Using a Hartmann-Shack aberrometer under low-light conditions (10 lx) and slit-lamp microscopy, Kamiya et al. [16] concluded that the surgical technique probably induced no significant change in pupil diameter and that pupil diameters were not significantly associated with the amount of vaulting. Another study evaluated pupil size before and 6 months after surgery under mesopic conditions (50 lx) using a WaveScan aberrometer [12]. In their 50-eye series implanted with a V4 model, pupil size decreased in 64% of eyes, increased in 20%, and remained unchanged in 16%. Overall, mesopic pupil size was significantly decreased after surgery (*P* = 0.01). Using a similar approach, Li et al. [17] studied highly myopic eyes and recorded larger decreases in pupil diameter after surgery in eyes with the lowest amounts of preoperative myopia.

Totsuka et al. [18], with a central-hole ICL (V4c model), evaluated pupillary dynamics using an infrared pupillometer. Their measurements revealed no significant differences between the scotopic pupil results before and after surgery. Recently, Zhu et al. [19] conducted a prospective observational study comparing dynamic pupillometry characteristics before and after implantation of two different ICL models: V4 (without a central port) and V4c (with a central port). A computerized automatic pupillometry system equipped with near-infrared illumination was used. Pupil diameter was proved to be smaller under mesopic and low photopic conditions; however, at 3 months after implantation, pupil diameter had recovered to its preoperative value under scotopic illumination. No significant differences were observed for eyes implanted with V4 and V4c ICLs.

All of the above-mentioned published studies agree that there either was no significant change or a decrease in pupil diameter (in mesopic or scotopic conditions) in the postoperative period. In our study however, we observed a tendency for pupil size to increase in all light conditions, although in relation to the postoperative vault reached.

Our study addresses pupil diameter measurement internally, without having to estimate what the actual diameter should be as a function of the corneal shape or the depth of the anterior chamber. In addition, it does so under fully reproducible ambient light conditions, lending more validity and consistency to our results. Furthermore, this mode of measurement allows us to verify the relationship between the diameter of the pupil and that of the ICL optic zone, which can undoubtedly have clinical repercussions. Although possible dysphotopic phenomena were not measured in the present study, with this approach we could preoperatively anticipate whether the optical zone of the intraocular lens under scotopic ambient light conditions could be exceeded. Consequently, we are able to warn patients about the possibility of halos for this reason. Moreover, and in line with our results, in case of disabling dysphotopsia related to pupillary enlargement derived from a high postoperative vault, it is possible to try to reduce this dysphotopsia by decreasing the vault either by rotating the pIOL vertically [22, 23] or by replacing the pIOL for a smaller one. Expected future improvements in formulas for pIOL sizing may enable a more accurate prediction of postoperative vault. This in turn would make it possible to aim for lower vaults in some eyes with larger scotopic pupils, and thus lead to a decreased scotopic pupil diameter and therefore likely a lower burden of associated dysphotopsias.

It is interesting to note that in our series, scotopic pupil exceeded the optical zone of the implanted ICL in more than half of the cases (60%). In approximately half of these eyes (46.15%), the scotopic pupil exceeded the optical zone of the lens on the nasal side, while in the other half exceeded it on both sides. The explanation is undoubtedly the physiological nasalization of the pupil. It is also noteworthy that the vault range was not correlated with the magnitude of change between the two pupillary states i.e., the pupillary dynamics do not relate to the dynamism of the vault. We also found no correlation with age, although it must be stressed that all patients were younger than 50 years.

Some authors have suggested the mechanical contact between the ICL and the posterior iris surface and/or the irritation of the uveal tissue as the cause of decreases in contraction amplitude and velocity of the pupil [17]; however, we do not agree with this statement. Eyes implanted with a pIOL do not show any signs of inflammation despite the iris continuously sliding and pushing the ICL down during pupillary movements. Conversely, after observing these pupillary movements with the dynamic AS-OCT device, we assume that changes in pupil diameter are mainly related to pIOL vault and the biometric characteristics of the anterior segment. The fact that the pupil diameter is larger in higher vaults suggests a simple issue of space: the more the pIOL protrudes, the more difficult it is for the iris to slide along its surface, and vice versa, in cases of low vault. However, this aphorism does not justify all cases. Both vault and pupillary dynamics are affected by iris configuration, thickness, and reactivity, as well as by how the lens is inserted and the crystalline lens’s own anatomy. Further studies will help us to discern the contribution of the different structures of the anterior segment to these changes in pupil diameter and vault changes.

We have considered a reasonable research time, four months on average and not exceeding one year, to study the postoperative pupil. Considering that we withdraw any postoperative treatment one week after surgery and that the patients are fully discharged from their operation 1 month after surgery, allowing them to resume a completely normal life, we believe that this time is enough for the results not to be influenced by the postoperative recovery. Moreover, the aim of this report was not to study the changes that the pupil will experience over time, which could be cause for future analysis.

Neither has our study attempted to relate scotopic pupillary diameter with the possible symptoms of dysphotopsia that this could cause while it could be interesting to do so in a future work. Lim et al. [12] found that halos were significantly related to the difference between mesopic pupil size and implantable collamer lens optic zone diameter, but clinical experience tells us that there is a very important subjective factor on how the neuroadaptation of patients occurs versus the objective fact that their pupils exceed the optic zone of the lens in scotopic conditions.

Finally, among the limitations of our study are the manual image acquisition and the short time that was left for our patients to adapt to the dark environment of the examination room. Also, a brighter light may possibly be needed to attain maximum miosis. However, we agree that these will not affect the validity of our results.

## Conclusions

In conclusion, pupil diameter may change after implantation of an ICL, and this change is related to the postoperative vault. Measurement techniques based on dynamic AS-OCT enables us to determine scotopic pupil diameter reliably and precisely in the iris plane before and after pIOL surgery. Further studies using this promising technology in larger series might provide more accurate data about pupil size changes after ICL surgery to better predict postoperative symptoms, and thus better counsel our patients. Meanwhile, the results of the current study favor the inclusion of pupil measurement in the preoperative assessment of candidates for implantation of a pIOL using non-invasive, easy-to-use, and precise pupillometry technique based on dynamic AS-OCT devices.

## Supplementary Information

**Additional file 1.** Assessment of changes in pupil diameter using swept-source anterior segment optical coherence tomography after phakic collamer lens implantation. The video shows the methodology used to measure the pupillary diameter before and after the implantation of an implantable collamer lens (ICL) with a central port (EVO + model) in different ambient light conditions using the CASIA2 anterior segment dynamic OCT.

## Data Availability

The dataset supporting the conclusions of this article is available from the corresponding author on reasonable request.
